# Review of Potential *Pseudomonas* Weaponry, Relevant to the *Pseudomonas–Aspergillus* Interplay, for the Mycology Community

**DOI:** 10.3390/jof6020081

**Published:** 2020-06-06

**Authors:** Paulami Chatterjee, Gabriele Sass, Wieslaw Swietnicki, David A. Stevens

**Affiliations:** 1 California Institute for Medical Research, San Jose, CA 95128, USA; chatterjee_paulami@yahoo.co.in (P.C.); Gabriele.Sass@cimr.org (G.S.); 2 Department of Immunology of Infectious Diseases, Hirszfeld Institute of Immunology and Experimental Therapy, Polish Academy of Sciences, 50-114 Wroclaw, Poland; wieslaw_swietnicki@hotmail.com; 3 Division of Infectious Diseases and Geographic Medicine, Department of Medicine, Stanford University School of Medicine, Stanford, CA 94305, USA

**Keywords:** *Pseudomonas aeruginosa*, *Aspergillus fumigatus*, *Pseudomonas–Aspergillus* interactions, cystic fibrosis, quorum sensing, virulence factors, quinolones, siderophores, phenazines, secretion system

## Abstract

*Pseudomonas aeruginosa* is one of the most prominent opportunistic bacteria in airways of cystic fibrosis patients and in immunocompromised patients. These bacteria share the same polymicrobial niche with other microbes, such as the opportunistic fungus *Aspergillus fumigatus.* Their inter-kingdom interactions and diverse exchange of secreted metabolites are responsible for how they both fare in competition for ecological niches. The outcomes of their contests likely determine persistent damage and degeneration of lung function. With a myriad of virulence factors and metabolites of promising antifungal activity, *P. aeruginosa* products or their derivatives may prove useful in prophylaxis and therapy against *A. fumigatus*. Quorum sensing underlies the primary virulence strategy of *P. aeruginosa*, which serves as cell–cell communication and ultimately leads to the production of multiple virulence factors. Understanding the quorum-sensing-related pathogenic mechanisms of *P. aeruginosa* is a first step for understanding intermicrobial competition. In this review, we provide a basic overview of some of the central virulence factors of *P. aeruginosa* that are regulated by quorum-sensing response pathways and briefly discuss the hitherto known antifungal properties of these virulence factors. This review also addresses the role of the bacterial secretion machinery regarding virulence factor secretion and maintenance of cell–cell communication.

## 1. Introduction

Intermicrobial competition between *Pseudomonas aeruginosa* and *Aspergillus fumigatus*, two ubiquitous microbes, occurs in environmental niches, such as soil and water. Researchers are most interested in how their interactions in the human biosphere may affect the propensity for disease, particularly in the immunocompromised host and in persons with cystic fibrosis [1,2,3,4]. In both the latter settings, both microbes, separately, or sometimes together, are responsible for considerable morbidity and mortality. For these reasons, researchers have for decades been studying interactions between these two microbes [5,6,7,8,9,10,11,12,13,14,15,16,17,18,19,20,21,22,23,24,25,26]. Interesting discoveries about their interactions have been made in recent years, by many laboratories. It is our expectation that in the future more mycologists will be attracted to these studies. We hope that this summary is a helpful introduction for mycologists who may be less familiar with the capabilities of *P. aeruginosa* that may affect intermicrobial competition and may pursue unraveling the mysteries and complexities of this inter-kingdom interaction. We can only summarize some of this knowledge here and cannot cover all the points (which we, and even some *Pseudomonas* researchers, are still learning) of *P. aeruginosa* physiology, genetics, and virulence that would be relevant to the *Pseudomonas* side of the equation.

## 2. Why *Pseudomonas aeruginosa*?

*P. aeruginosa* is one of the most prominent opportunistic bacteria found in the airways of persons with cystic fibrosis and in neutropenic patients [1]. It is a ubiquitous bacterium, commonly present in the environment, especially in soil and water, but also frequently present in burn wounds and surgical wounds [27,28,29]. These motile, rod-shaped, Gram-negative bacteria are also known to be associated with acute and chronic stages of lung disorders. It is widely recognized that this organism is responsible for the inflammatory exacerbation, progressive lung damage, and high morbidity and mortality rates seen in cystic fibrosis patients [30,31,32,33]. In those microenvironments, there is a constant battle for nutrients between *P. aeruginosa* and *A. fumigatus*, and the result of this competition ultimately determines inter-pathogen or host–pathogen relationships [34,35,36,37]. Depending on the host environment and nutrient availability, *P. aeruginosa* secretes several virulence factors, such as acyl-homoserine lactones, alkyl quinolones, rhamnolipids, phenazines, siderophores, which interact and interfere with the behavior of other microbes, including *A. fumigatus* [8,9,12,38,39,40,41,42,43,44], and facilitate survival of the bacteria. Some of these molecules, such as quorum-sensing factors, are used by *P. aeruginosa* for its cell-to-cell communication to facilitate survival strategies that benefit a group as a whole. It is also established that *P. aeruginosa* commonly undergoes a transition in cystic fibrosis patients, to a mucoid variant from a non-mucoid phenotype [1,45,46]. Transition into the mucoid variant is believed to be part of the remarkable survival strategies of *P. aeruginosa*, which helps the bacteria to adapt within the host during the chronic stages of infection. Non-mucoid to mucoid transition also facilitates biofilm formation, which adds to the repertoire of bacterial antifungal resistance, resistance to host immunity, and persistence of infection [46,47]. The highly infectious and virulent characteristics of *P. aeruginosa* are attributed to its strikingly large genome (~6.3 million base pairs) and a distinctly large proportion of regulatory genes [48]. With this effort to unravel different virulence strategies of *P. aeruginosa*, in this review we are aiming to provide a basic overview of key virulence factors of *P. aeruginosa* that are regulated by quorum-sensing response pathways and briefly discuss the hitherto known antifungal properties of these virulence factors.

## 3. Quorum Sensing and *P. aeruginosa*

One of the prominent features of *P. aeruginosa* is the extent of its “quorum-sensing” machinery, which enables the bacteria to readily form multicellular communities based on cell proximity, cell population density, and the extracellular environment [49,50,51]. Quorum sensing is a type of cell signaling mechanism that regulates bacterial gene expression and also initiates the production of multiple virulence factors [51]. Thus, quorum sensing benefits the bacterial population by imparting them with characteristics for survival.

**Figure 1:**
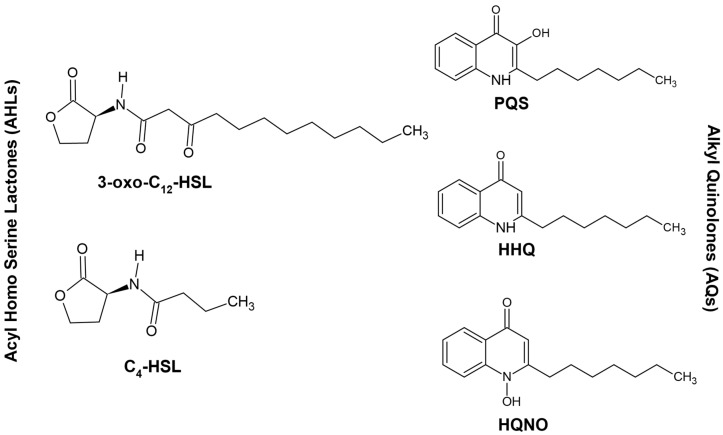
Structures of some common quorum signaling molecules of *Pseudomonas aeruginosa*.

The important quorum-sensing signal molecules in *P. aeruginosa* are acyl-homoserine lactones (AHLs) and alkyl quinolones (AQs). AHLs primarily include 3-oxo-C_12_-HSL and C_4_-HSL (Figure 1) and AQs include 2-heptyl-3-hydroxy-4-quinolone, otherwise known as *Pseudomonas* quinolone signal (PQS), its precursor 2-heptyl-4-quinolone (HHQ), and 2-heptyl-4-hydroxyquinoline-*N*-oxide (HQNO) (Figure 1) [52,53,54,55,56].

## 4. Interplay between Different Quorum Signaling Molecules and the Role of LasI/RhlI Systems in the Quorum-Sensing Circuitry

In *P. aeruginosa*, virulence characteristics and biofilm formation are controlled by the simultaneous interaction between three different quorum-sensing systems. The LasI and RhlI synthase catalyzes the production of the AHLs signals. AHLs act as auto-inducers that regulate the activity of the two transcriptional regulators, LasR and RhlR. 3-oxo-C_12_-HSL, a product of LasI, acts as a cognate ligand activating LasR, which then directs the transcription of several target genes, including *rhlR*, whose product synthesizes a second quorum-sensing signal, C_4_-HSL [41,49,53,57,58,59]. In turn, C_4_-HSL induces the transcriptional regulator RhlR, which activates the transcription of a different subset of target genes. Both LasR and RhlR contrastingly regulate the expression of the proteins PqsA, PqsB, PqsC, PqsD, PqsE, PqsH, and the transcriptional regulator PqsR (also known as MvfR, for multiple virulence factor regulator), which then leads to the production of the AQs, including HHQ and PQS [53,57,58,59]. The latter two AQs act as the cognate ligands inducing the activity of PqsR [60]. Thus, conventionally, interaction between the Las and Rhl systems is considered to form a hierarchical cascade, where the Las system is the starting point of the whole quorum-sensing circuitry [53,57,59,61]. The RhlR + C_4_-HSL targets include several genes directing the production of metabolites acting as virulence determinants, such as the *phzA1B1C1D1E1F1G1* and *phzA2B2C2D2E2F2G2* operons, *phzS*, *phzM*, *phzH* genes (all coding for a range of phenazines including pyocyanin), the *rhlAB* operon and *rhlC* gene (rhamnolipids), the *hcnABC* operon (hydrogen cyanide), *lecA*, and *lecB* (two lectins), and *chiC* (chitinase) [38,59,62,63,64,65]. Since LasR activates the Rhl system, it could be expected that disruption of *lasR* would prevent the expression of both LasR and RhlR-regulated target genes. However, it was found that MvfR and RhlR-regulated functions are still produced in *lasR* mutants under certain conditions [66,67,68]. For instance, HHQ and pyocyanin are overproduced by *lasR* mutants in the late stationary phase [53,68]. This observation paved the way for the hypothesis that quorum sensing in *P. aeruginosa* may not always be hierarchical and that the three quorum-sensing systems may be activated independently [68,69]. Indeed, Dekimpe and Déziel found that activation of the RhlI system is only delayed but not blocked in a *lasR* mutant, thus allowing the expression of multiple virulence factors during the late stationary phase [69]. These authors also reported that, in this situation, RhlR may overcome the absence of the Las system by activating LasR-controlled functions, including the production of 3-oxo-C_12_-HSL and PQS [69]. Thus, *P. aeruginosa* can bypass the unavailability of one of its primary quorum-sensing systems by initiating a far more complex pathway of quorum-sensing regulation. Accordingly, several LasR-defective strains retaining functional RhlR systems have been reported among CF isolates [70]. A study by Lee et al. reported the identification of a fourth quorum-sensing signal molecule IQS (2-(2-hydroxyphenyl)-thiazole-4-carbaldehyde), which was named for its role in integrating quorum-sensing network and stress response in *P. aeruginosa* [71]. The authors reported that IQS synthesis is tightly regulated by *las* under phosphate-replete conditions (phosphate-replete *Pseudomonas* medium P) but is also activated by phosphate limitation [71]. However, a recent commentary has addressed the fact that the structure of IQS is identical to that of aeruginaldehyde [72], which could be either a by-product of the siderophore pyochelin synthesis pathway [73] or a pyochelin metabolite [74]. The author also reported that there is controversy regarding its biosynthesis from *ambABCDE* genes, a conclusion that had been reported previously [72].

**Figure 2:**
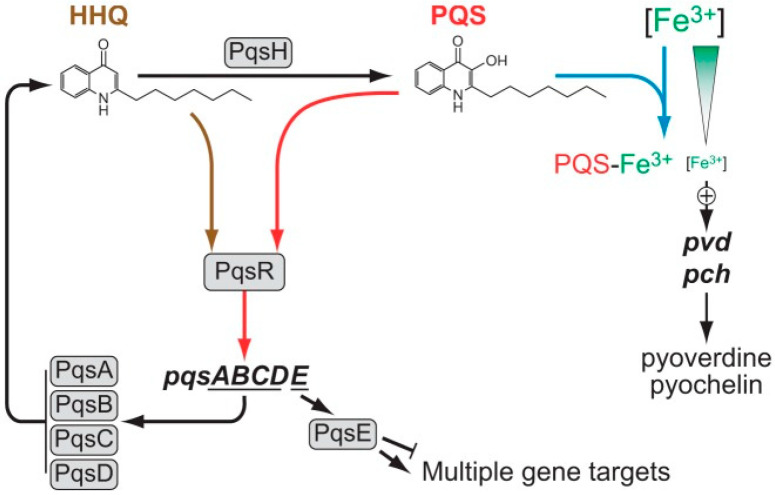
AQ-dependent quorum-sensing pathway in *P. aeruginosa*—transformation of HHQ to PQS. Action of the PqsABCD proteins result in HHQ, which is converted to PQS by PqsH. Autoinduction occurs when either HHQ or PQS binds to PqsR and amplifies the expression of the *pqsABCDE* operon. PqsE is a putative metallohydrolase of unsettled enzymatic function targeting multiple downstream genes; its target appears to be the function of RhlR. Later studies have revealed the role of PqsE in HHQ biosynthesis. PqsE acts as thioesterase, hydrolyzing the biosynthetic intermediate 2-aminobenzoylacetyl-coenzyme A to form 2-aminobenzoylacetate, the precursor of HHQ [80]. PQS has additional functionalities as a potent iron chelator, which induces expression of the *P. aeruginosa* siderophores (pyoverdine and pyochelin). Filled arrows represent positive regulation, whereas blunted lines represent negative regulation [75]. AQ—alkyl quinolone, HHQ—2-heptyl-4-quinolone, PQS—*Pseudomonas* quinolone signal. Figure reproduced from Ilangovan et al., 2013 [75].

Biosynthesis of the primary AQ PQS starts with the conversion of anthranilate (which originates via two anthranilate synthases, PhnAB and TrpEG) into HHQ (Figure 2). This conversion is mediated by the enzymes encoded within the five gene operon *pqsABCDE*. HHQ is finally converted into PQS by the PqsH monooxygenase (Figure 2). HHQ and PQS are autoinducers of MvfR, which when bound to a ligand, is activated and induces the transcription of *pqsABCDE* and *phnAB* operons, thus increasing the levels of PQS (Figure 2) [50,57,75,76,77,78]. MvfR cannot by itself regulate the targets. The regulator of *phz*, *hcn*, *chiC*, *lecA*, and others is PqsE, which acts in concert with RhlR [79]. This AQ-mediated quorum sensing is controlled by LasR and RhlR, where LasR positively regulates the transcription of *pqsR*, *pqsABCDE*, and *pqsH* whereas RhlR negatively regulates *pqsR* [75]. Déziel et al. reported that the regulator MvfR (PqsR) plays a central role in the entire AQ-dependent quorum-sensing cascade of *P. aeruginosa* [67]. In short, MvfR controls the expression of *phnAB* that catalyzes the synthesis of anthranilic acid or anthranilate. It also controls the transcription of *pqsA-E* genes, which are primarily responsible for the synthesis of AQ HHQ, the precursor of PQS [67].

## 5. Involvement of Quorum-Sensing Molecules in *P. aeruginosa* Virulence and Antifungal Activity

Products of *P. aeruginosa* quorum-sensing mutants (PA14Δ*lasR*-*rhlR*- and PA14Δ*rhlR*) were found to have fewer inhibitory effects on *A. fumigatus* biofilms compared to those of their wild-type strain [23]. This loss of antifungal activity in quorum-sensing mutants was attributed to a lack of pyoverdine production [23]. It is established that AHLs serve as important virulence factors inducing inflammation and acting as signaling molecules, promoting intra- and interspecies communication in *P. aeruginosa* [69,81,82]. AHLs are also reported to possess antifungal activity. The inhibitory effects of AHLs on *A. fumigatus* growth have been studied [8]. Using *P. aeruginosa* quorum-sensing mutants (PAO1Δ*lasI* and PAO1Δ*lasR*), it had been suggested that 3-oxo-C_12_-HSLs may inhibit biofilm formation in *A. fumigatus*. AQs, especially PQS, are widely studied for their role in *P. aeruginosa* virulence. One previous study reported that PQS and its precursor HHQ both possess the ability to alter *A. fumigatus* biofilm biomass and hyphal structures [17]. In this study, the authors prepared different derivatives of the quinolone framework and documented significant changes in the ability of these molecules to inhibit fungal biofilm formation, thus demonstrating the importance of the anthranilate ring in antibiofilm activity [17]. PQS upregulates an array of downstream virulence genes, including the redox-active phenazines, which have been studied extensively for their inhibitory effects against *A. fumigatus* growth [9]. Since *P. aeruginosa* and *A. fumigatus* co-exist in the same clinical and environmental niches, these interactions and molecular cross-talks are likely important for their inter-kingdom relationship. Studies have also identified the role of PQS in iron chelation [83,84]. PQS has a high affinity for ferric iron (Fe^3+^) and by binding to iron it reduces the availability of free iron in the environment. The addition of exogenous PQS induces the upregulation of genes responsible for siderophore (pyoverdine, pyochelin) production (Figure 2) [83,85,86,87]; these efficiently scavenge accessible iron under iron-deficient conditions. PQS may function as an iron trap and storage molecule in cell membranes, and it could transport iron to the bacterial cells [84]. However, Diggle et al. reported that the explanation of siderophore gene regulation is likely insufficient [85]. Rampioni et al. suggested that the expression of siderophore genes in *P. aeruginosa* is also regulated by PqsE, which further suggests that iron trapping by PQS does not solely control the siderophore production by *P. aeruginosa* [88]. Interestingly, a recent study by our group has revealed that PQS may play a dual role in response to environmental iron; under high-iron conditions PQS may interact with *A. fumigatus* siderophores and provide essential iron to the fungus, whereas, under low-iron conditions PQS is toxic to the fungus [26]. This study also reported that PQS induced *A. fumigatus* conidiation in a high-iron environment where PQS + iron caused higher fungal conidiation than the iron alone. Conversely, PQS inhibited *A. fumigatus* via chelation under low-iron conditions. Thus, this study illustrated a complex and bi-directional nature of the interplay between these two microbes [26].

## 6. Siderophores in *P. aeruginosa* and Their Anti-*Aspergillus* Effects

In order to sequester and utilize extracellular iron, *P. aeruginosa* secretes siderophores, which are low-molecular-weight iron-chelating agents, highly efficient in binding available iron (essential for bacterial growth) [89]. These siderophore–iron complexes are recognized by the bacteria, which then internalize the iron [89]. It has been established that these siderophores are generally microbe specific. *Pseudomonas* siderophores block iron utilization by the fungus and *Aspergillus* siderophores block iron utilization by the bacteria [25]. In mammalian hosts, iron is tightly bound to iron-chelator proteins such as hemoglobin, transferrin, lactoferrin, and ferritin. During infection, the host immune system sequesters iron (by employing the aforementioned iron chelators) to limit free iron, thus inhibiting microbial growth and further preventing bacterial infection. Bacteria, by employing siderophores, scavenge iron and counter this iron-limitation approach of the host. *P. aeruginosa* produces two siderophores, pyoverdine and pyochelin, that are important in both biofilm formation and bacterial virulence [34,42].

The major siderophore produced in *P. aeruginosa* is pyoverdine, which is essential for establishing infection in the cystic fibrosis lung [34]. One previous study identified that *P. aeruginosa* inhibited *A. fumigatus* growth through iron starvation, which was thought to be mediated by metabolites from the bacterium that modulated the siderophore production in the fungus [90]. Our recent study showed that *P. aeruginosa* pyoverdine can suppress *A. fumigatus* growth and biofilm formation via the chelation of iron, reducing its availability to the fungus, in a low-iron environment [23]. Using several *P. aeruginosa* deletion mutant strains, defective in the expression of important virulence factors, this study evaluated the antifungal activity of the mutants (and their extracellular products) against *A. fumigatus*. It was found that siderophore pyoverdine mutant (PA14Δ*pvdD*-*pchE* and PA14Δ*pvdD*) supernatants showed less inhibition of *A. fumigatus* growth and biofilm formation, compared to wild-type *P. aeruginosa* strain supernatants [23]. Moreover, the inhibitory effect of pyoverdine-deletion mutants was restored by pure pyoverdine. Clinical *P. aeruginosa* isolates derived from the lungs of cystic fibrosis patients revealed a correlation between the amount of pyoverdine produced and the antifungal activity of clinical isolates [23]. The results suggest the siderophore pyoverdine is a crucial factor in *P. aeruginosa* antifungal activity under low-iron conditions [23]. Even under hypoxic conditions, pyoverdine was identified as the principal mediator of antifungal activity on both forming and preformed fungal biofilms [23]. This study also demonstrated that pyoverdine leads to iron starvation and results in an increase of siderophore secretion by *A. fumigatus*. Concomitantly, an *A. fumigatus* siderophore mutant was found to be hyper-susceptible to *P. aeruginosa* pyoverdine [23]. Another related study further sheds light on the pyoverdine-mediated antifungal activity of *P. aeruginosa* [25]. This study reported that *P. aeruginosa* supernatants produced in the presence of wild-type *A. fumigatus* planktonic supernatants showed reduced activity against *A. fumigatus* biofilms despite higher production of pyoverdine by *P. aeruginosa* [25]. We proposed that *A. fumigatus* siderophores can defend the fungus against antifungal *P. aeruginosa* effects by scavenging iron, thus, supporting the fungus and resulting in denial of iron to the bacteria [25]. Thus, key aspects of the competition between *P. aeruginosa* and *A. fumigatus* were found to be the relative amounts of siderophores produced, the relative affinity for Fe [23], and likely the timing of the siderophore production.

Pyochelin, the second siderophore of *P. aeruginosa*, is structurally different from pyoverdine. It has a lower molecular mass than any other bacterial or fungal siderophores [36]. Unlike pyoverdine, it does not contain catecholate or hydroxamate groups [91], and it is likely a redox-active compound, being able to impart oxidative stress to human immune cells and bacteria [92,93]. Our laboratory has recently indicated pyochelin activity against another eukaryote, *Trypanosoma cruzi* [94]. Interestingly, pyochelin has also been reported to possess a high affinity for zinc [95]. Briard et al. demonstrated two antifungal effects of pyochelin on *A. fumigatus* cells, with inhibition resulting from iron and zinc deprivation from the medium, as well as induction of oxidative stress, mediated by reactive oxygen species (ROS) and reactive nitrogen species (RNS) [93]. The authors also identified the possible role of pyochelin as an external iron chelator for fungal cells, thus stimulating fungal growth and promoting survival when iron access is limited [93].

Competition by *A. fumigatus* for iron via *A. fumigatus* siderophores is likely not the only weapon available to *A. fumigatus* in its battles with *P. aeruginosa*. Moreover, *A. fumigatus* can also influence *P. aeruginosa* in ways that are favorable for *P. aeruginosa* [96]. We have cited how each of the pathogens can make modifications in the chemical products of the other, in such a way that the behavior of one or both of them can be affected [9]. Details about the influences of *A. fumigatus* on *P. aeruginosa* are outside the scope of the present review, but this is an important topic that demands further research and a review of the evidence.

Moreover, most of the research to date on the *P. aeruginosa*–*A. fumigatus* interaction has focused on molecules that can diffuse in a liquid environment. However, both *P. aeruginosa* [97,98,99,100,101,102,103,104,105] and *A. fumigatus* [106,107,108] are known to produce volatiles, usually small lipophilic organic molecules, and these represent other potential weapons, which might be particularly important in the lung environment. Studies have indicated some *P. aeruginosa* molecules have the potential to inadvertently benefit the economy of their rival [109]. In our studies (unpublished), we find *P. aeruginosa* volatiles inhibitory to *A. fumigatus*, and have identified some candidate molecules to explain this.

## 7. Elastase in *P. aeruginosa* and Antifungal Effects

*P. aeruginosa* can produce different enzymes and toxins as extracellular virulence factors, which include proteases, lipase, exotoxin A, etc. [110,111]. All of these factors increase the pathogenicity and survival rates of the bacteria in the environments where they must compete [41,43,44]. Among the different proteases secreted by *P. aeruginosa*, the most well-studied and well-characterized protease is the elastase (LasB), which catabolizes the substrate elastin [112,113]. LasB is secreted by non-mucoid *P. aeruginosa* variants, and its secretion is reported to be reduced in mucoid variants isolated in the chronic stages of cystic fibrosis disease progression [114]. LasB hydrolyzes proteins of the extracellular matrix and degrades cells of the host immune response, thus exerting tissue-damaging activity during the infection [13,110,112]. The biosynthesis of LasB is regulated by the quorum-sensing regulators LasR and RhlR [62]. LasB has been studied for its role in promoting microbial infection in cystic fibrosis. Smith and colleagues have reported that, in the presence of the fungus *A. fumigatus*, the production of LasB was increased [13]. In this co-culture experiment, 60% of *P. aeruginosa* isolates produced significantly higher amounts of LasB in the presence of the fungus than in its absence, and the expression of the elastase-producing gene *lasB* was also increased [13]. This study identifies elastase as a potentially important contributor to co-colonization with these two microbes (*P. aeruginosa* and *A. fumigatus*) in cystic fibrosis lung infection.

## 8. Rhamnolipids in *P. aeruginosa* and Their Antifungal Effects

**Figure 3:**
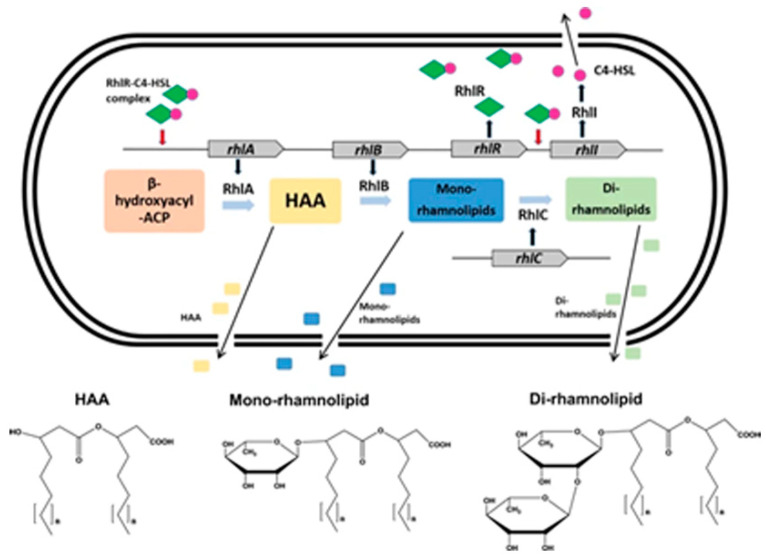
Rhamnolipid pathway in *P. aeruginosa* and its regulation by Rhl system. RhlR (green diamonds) binds with C_4_-HSL (magenta circles) produced by RhlI to form a RhlR–C_4_-HSL complex. RhlR–C_4_-HSL complex interacts with the rhlA promoter to initiate transcription of the rhlAB genes to produce rhamnolipids. HAA is 3-(hydroxyalkanoyloxy) alkanoic acid, which is converted into mono, and later di-rhamnolipids. Other studies have indicated LasR, and not RhlR, activate *rhlI* transcription; and that sometimes the precursor of RhlA is not 3-hydroxy-acyl-ACP (may be activated in CoA). Figure reproduced from Wood TL et al., 2018 [115].

Rhamnolipids are a class of secondary metabolites secreted by *P. aeruginosa* that are also known to possess virulence properties. Rhamnolipid biosynthesis pathways are regulated by quorum sensing. Products of *rhlAB* operon and *rhlC* gene catalyze the three final steps of the rhamnolipid biosynthesis pathway (Figure 3) [12,115]. *P. aeruginosa* can produce both mono- and di-rhamnolipids. These glycosidic exoproducts are reported to be overproduced during stress conditions [12]. Such conditions may apply to concurrently occupied microbial niches.

Rhamnolipids are surface-activated molecules involved in the promotion of bacterial swarming motility [116,117] facilitating the detachment of cells from *P. aeruginosa* biofilms, especially from the center of microcolonies [118,119,120]. Rhamnolipids have antimicrobial properties against diverse Gram-positive and Gram-negative bacteria [121]. Rhamnolipids intercalate into the plasma membranes of other bacteria and induce disorganization of the plasma membrane [122]. Previous studies have also reported antifungal properties of *P. aeruginosa* rhamnolipids on a range of fungal species including *Candida albicans*, *Fusarium oxysporum*, and A. fumigatus [19,56,123,124,125]. The most prominent effect of di-rhamnolipids on *A. fumigatus* is believed to be hyphal wall thickening and altered development of the fungal hyphae with short ramifications at the tip [19,93]. It is also reported that the mode of action of di-rhamnolipids is not fungicidal but fungistatic [19]. Briard et al. described that *P. aeruginosa* di-rhamnolipids strongly bind to the fungal hyphae with the help of extracellular matrix (ECM) polysaccharide galactosaminogalactan (GAG) and induce the production of GAG and melanin in the ECM [19]. Simultaneously, di-rhamnolipids inhibit β-1,3-glucan synthase at the hyphal tip, which promotes formation of new apices, containing active β-1,3-glucan synthase [19,93]. This new β-1,3-glucan synthase is further inhibited by the rhamnolipids, thus inducing the multibranched hyphal phenotype (with short apical cells) in the fungus [19,93]. Di-rhamnolipids also increase chitin synthesis in *A. fumigatus*, which serves as a compensation for β-1,3-glucan synthase inhibition. Thus, rhamnolipids modify the hyphal phenotype and nature of the ECM surrounding the hyphae [19].

## 9. Phenazines and Their Antifungal Role in *P. aeruginosa*

Phenazines are a group of nitrogen-containing heterocyclic compounds produced as secondary metabolites by a diverse range of bacteria, including *P. aeruginosa*. Phenazines possess distinct physicochemical properties, including bright pigmentation, redox properties, and the ability to change color with pH and redox state [126]. In *P. aeruginosa*, phenazine production is triggered by a cascade of the quorum-sensing response, affected by high cell density, environmental signals, and intracellular physiology of the bacteria. Phenazines play multiple roles in contributing to the behavior, growth, survival, and ecological fitness of the producing bacterium [41,127]. Depending on the environmental signals, phenazines alter intracellular electron flow patterns and balance the redox state within the bacteria [12]. They also serve important roles in bacterial biofilm formation and modify cellular architecture [127,128]. In contrast, in non-producers (competing microbes or the human host), phenazines exert numerous effects, including morphological changes, reactive oxygen species (ROS) and reactive nitrogen species (RNS)-mediated inflammation and cell damage, and inhibition of significant genes, thus modifying host cellular responses [127,128].

**Figure 4:**
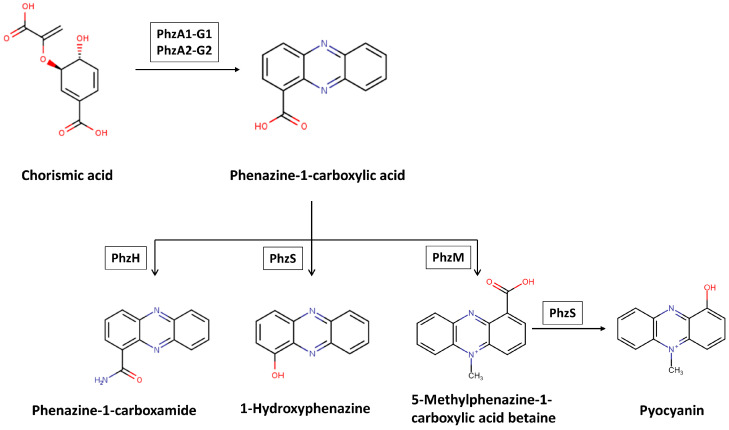
Phenazine pathway in *P. aeruginosa.*

*P. aeruginosa* produces multiple phenazine derivatives during growth and in response to environmental factors. Four important phenazines produced by *P. aeruginosa* are phenazine-1-carboxylic acid (PCA), pyocyanin, 1-hydroxy phenazine (1-HP), phenazine-1-carboxamide or PCN, each of which has distinct chemical properties (Figure 4) [41,129,130]. Genes encoding the enzymes of the phenazine biosynthesis pathway are found in two redundant seven-gene operons. These operons, *phzA1B1C1D1E1F1G1* (*phz1*) and *phzA2B2C2D2E2F2G2* (*phz2*) encode two sets of proteins that catalyze the synthesis of PCA, the precursor for all other phenazine derivatives (Figure 4) [130]. Conversion of PCA to PCN is mediated by the enzyme PhzH and conversion of PCA to pyocyanin is mediated by the enzymes PhzM and PhzS. Both conversions are influenced by the surrounding environment. It is reported that, in planktonic cultures, the majority of PCA modification leads to the production of pyocyanin. In contrast, in biofilms, a substantial amount is converted to PCN [130]. Although the *phz1* and *phz2* operons have significant similarity (~98% similarity), they are differentially regulated; *phz1* is regulated by the quorum-sensing response, whereas the factors controlling *phz2* expression have not yet been identified [129,130,131,132].

Pyocyanin, one of the *P. aeruginosa* phenazines, has been identified in cystic fibrosis patient sputum at concentrations up to 135 µM [133]. High pyocyanin concentrations are believed to be responsible for the persistent lung damage observed in those patients [134]. *P. aeruginosa* phenazines are reported to exert fungicidal effects on *A. fumigatus*. Phenazines can diffuse across the cell membranes and act as reducing agents, causing uncoupling of oxidative phosphorylation and generating toxic intracellular ROS that are harmful to the fungus [135,136]. A remarkable study by Moree and colleagues used a mass-spectrometry-based metabolomics approach to provide insight into the interspecies interaction between *P. aeruginosa* and *A. fumigatus* at the molecular level [9]. They demonstrated that bacterial phenazine metabolites were altered by fungal molecules to support the fungus in the polymicrobial communities. Interestingly, this study identified that the primary phenazine metabolite secreted by *P. aeruginosa*, PCA, was converted into 1-HP, which was further transformed into phenazine-1-sulphate (PS) and 1-methoxyphenazine (1MP) [9]. These phenazine metabolites were reported to have diverse effects on the fungus: whereas 1-HP and 1MP showed inhibitory effects on fungal growth compared to PCA, 1-HP also enhanced the fungal siderophore production and PS showed no antifungal activity [9]. The exact roles of these intermediate metabolites on the fungus are yet to be determined. *A. fumigatus* also mediated the conversion of PCA into pyocyanin and promoted the production of mixed phenazine dimers [9]. The exact cause for the facilitation of phenazine dimer production is still not known but it could be a defense mechanism for the fungus. This bioconversion may lower the concentration of pyocyanin, thus possibly affecting the quorum-sensing pathway and subsequent virulence factor production by *P. aeruginosa* [9]. Briard et al. identified inhibitory effects of *P. aeruginosa* phenazines (pyocyanin, PCA, PCN, and 1-HP) on *A. fumigatus* mitochondrial architecture and hyphal growth where the primary mode of inhibition was ROS–RNS-mediated oxidative stress [12]. This study showed that at high phenazine concentrations, phenazines, specially pyocyanin and PCA, can penetrate fungal cells (conidia and hyphae) and affect production of mitochondrial superoxide dismutase. A lack of this enzyme leads to increase in ROS–RNS production in the mitochondria and subsequent ROS release in the cytoplasm that ultimately leads to fungal death. The authors also reported that 1-HP acts as an iron chelator that represses fungal growth in low-iron environments [12]. Interestingly, some studies also reported a possible dual signaling role of phenazine-derived metabolites on fungal biofilm formation and fungal development [136,137]. Zheng et al. reported that bacterial phenazine production can differentially modulate biofilm formation by *A. fumigatus*; whereas low-to-moderate levels of ROS can act as a sporulation signal in fungal development, higher levels of ROS can act as a toxic substance [137]. Thus, *A. fumigatus* development switches from vegetative growth to conidiation in response to the phenazine gradient in the co-culture medium, correlating with levels of phenazine radicals produced in the phenazine-mediated redox cycles [137,138]. Our recent study, presented at AAAM9 (9th Advances Against Aspergillosis and Mucormycosis), identified mechanisms of *P. aeruginosa* that enables the bacteria to switch from iron-denial-based (pyoverdine-based) to toxin-based (pyocyanin-based) antifungal activity in response to environmental iron concentrations [139]. We found that under high-iron conditions *P. aeruginosa* exerted phenazine pyocyanin-based antifungal mechanisms to compete with *A. fumigatus* [139]. In this study, we demonstrated that the addition of iron (in the form of either FeCl_3_ or blood) led to the reduction of pyoverdine content while increasing the phenazine content in bacterial supernatants. Subsequent experiments revealed strong antifungal activity of these supernatants against *A. fumigatus* biofilms. Similarly, *P. aeruginosa* supernatants not containing pyocyanin were significantly less antifungal toward *A. fumigatus* biofilms under high-iron conditions. We also found that pure pyocyanin at concentrations over 125 µM interfered with fungal metabolism [139]. Therefore, under high-iron conditions, pyocyanin appears to replace the antifungal activity of pyoverdine, observed under low-iron conditions.

## 10. Role of Secretion Systems in *P. aeruginosa* Virulence and Quorum-Sensing Response

Like other Gram-negative bacteria, *P. aeruginosa* possesses an array of secretion systems (Types I–VI), which are distinguished by the characteristics of components forming the secretion machinery [140]. These secretory machineries are utilized in an orchestrated way to deliver virulence factors either to the extracellular environment or directly into other cells. These secretion systems are just beginning to be explored with respect to bacterial–fungal interactions. The heme-binding protein HasAp from *P. aeruginosa*, important for iron acquisition, is an example of a protein secreted through the Type I Secretion System (T1SS) [141,142]. Secretory enzymes and toxins with remarkable virulence properties, such as elastase (LasB), lipase (LipA), alkaline phosphatase (PhoA), phospholipase C (PlcH), and exotoxin A (ExoA) are secreted through the Type II Secretion System (T2SS) [140]. The Type III secretion system (T3SS) is already established as a major virulence factor in *P. aeruginosa* that contributes to cytotoxicity and acute infections [140,143,144], and this cytotoxicity is considered to be associated with the activity of effector proteins that are secreted in a T3SS-dependent manner. The already known effectors in *P. aeruginosa* identified with T3SS are ExoS, ExoT, ExoU, and ExoY [140]. Among these effectors, ExoU is reported to be the most potent cytotoxin, with phospholipase activity, contributing to lung tissue damage in cystic fibrosis patients [145]. *P. aeruginosa* contains one or more genomic island-associated Type IV secretion systems (T4SS) that play a crucial role in horizontal gene transfer of integrative and conjugative elements [146], and the proteins secreted through the T4SS are reported to be associated with antibiotic resistance to the bacteria [142]. Type V secretion system (T5SS) secretes virulence factors and mediates cell-to-cell adhesion and biofilm formation [142]. A substrate example for the T5SS in *P. aeruginosa* is EstA protein, which has esterase activity and is involved in the production of rhamnolipids, cell motility, and biofilm formation [142,147]. The Type VI Secretion System (T6SS) is described as the most important secretion system in maintaining bacterial cell–cell communication and is also implicated in bacterial virulence. *P. aeruginosa* harbors three Type VI secretion (T6S) loci, hemolysin co-regulated protein secretion island-I, II, and III (HSI-I, HSI-II, and HSI-III). Two major known substrates of T6SS in *P. aeruginosa* are hemolysin-co-regulated protein (Hcp) and valine–glycine repeat protein G (VgrGs) [142]. Quorum sensing differentially regulates the expression of genes at all three loci [148]. While quorum-sensing regulators, LasR and MvfR, negatively regulate HSI-I-associated genes, they positively regulate both HSI-II and HSI-III associated genes [148]. Another quorum-sensing enzyme, PqsE, is also reported to be required for the expression of part of HSI-III but not HSI-II, and inhibitors of the alkyl quinolone biosynthesis pathway are shown to downregulate HSI-II and -III gene expression [148]. It is reported that cell-cell communication and environmental signals play crucial roles in activating T6SS [149,150]. Gallique et al. described interesting modes of T6SS behavior, i.e., defensive and offensive modes [150]. They demonstrated that *P. aeruginosa* can perceive T6SS-mediated aggression of neighboring cells [150,151] and recognize T6SS attacks coming from other microbial communities [152]. The recognition and response in T6SS are mediated by the GacS–GacA signaling cascade, which is dependent on the balance between the hybrid sensor kinases RetS and LadS [150]. T6SSs in *P. aeruginosa* were originally identified in the PAO1 strain and later studied in the reference strain PA14 [153]. Studies have identified differences between these strains at the HSI-II and HSI-III loci, which further indicates the possible difference in virulence mechanisms in these two strains [148]. The HSI-I and HSI-II loci have been implicated in *P. aeruginosa*-mediated pathogenesis in progressive lung diseases [153]; HSI-III has also been identified as an essential component for bacterial virulence [148]. Recent studies have identified fungal-specific T6SS effector proteins (Tfe1 and Tfe2) in another Gram-negative opportunistic bacteria *Serratia marcescens* [154,155]. These effector proteins contributed to T6SS-mediated inhibition of fungal growth in the fungal species *Candida albicans*, *Candida glabrata*, and *Saccharomyces cerevisiae* [154,155]. Effector-based antifungal activity was also reported for *P. aeruginosa* T6SS protein Tse2, which showed toxic effects and inhibition of growth in the yeast *Saccharomyces cerevisiae* [156]. To evaluate the importance of T6SS, another recent study has followed a proteomic-based approach to study the abundance of the key T6SS components in *P. aeruginosa* [157]. These studies further implicate the *Pseudomonas* T6SS as a potential antifungal secretion machinery and a target for future therapeutic studies. Secretory systems of *P. aeruginosa* could also be a target of therapeutic approaches to bacterial control [158]. The bacterial pathogen is destructive to human tissues when it can penetrate the epithelial barrier.

Numerous research approaches are being pursued to develop *P. aeruginosa* anti-infectives that can inhibit the growth and/or reduce the virulence of the pathogen [159]. The strategies primarily involve development of novel compounds that impede *P. aeruginosa* biofilms to restore susceptibility toward antibiotics [159]. Targeting the quorum-sensing system with drugs is a novel approach to reducing *Pseudomonas* pathogenicity and thus infection [160,161]. As classic strategies to combat *Pseudomonas* infections have not been very successful, owing to such factors as development of resistance to antimicrobials, adverse drug effects on the host, or undesirable drug–drug interactions, combination therapy (including antibiotics and antivirulence drugs) and alternative approaches have been proposed [159,162]. These strategies targeted T3SS predominantly [163] and T2SS secondarily. Strategies to block T3SS utilized mainly small molecules as the pathogen is quickly internalized by the host cells under permissive conditions [164]. The approaches targeted function of the secreted proteins [165,166], assembly of the system [167], protein transport [168,169,170], and the bacterial ATPase PscN [171]. Blockage was also tried using a random screen of compounds [172] or through the rational design of dual-action small molecules [171] targeting the T3SS PscN ATPase (in silico) and the secretion of the T2SS effectors (experimental). These strategies, aimed at inhibiting *P. aeruginosa* pathogenesis in the mammalian host, should be explored to examine effects on *P. aeruginosa* intermicrobial interactions, in order to evaluate the roles of these secretions systems as the as yet unquantified components of *P. aeruginosa* antifungal weaponry.

## 11. In Vivo Interactions between *P. aeruginosa* and *A. fumigatus*

The effect of the *Pseudomonas*–*Aspergillus* interactions we have detailed must be translated into the in vivo effects. As our review here focuses on the in vitro and molecular mechanisms of *Pseudomonas* pathogenicity, a more extensive review of the in vivo effects is required and awaited. Animal models would suggest co-infection in the lungs with *P. aeruginosa* and *A. fumigatus* in leukopenic mice is a deadly combination [173]. The interaction between *P. aeruginosa* and *A. fumigatus* has received scant attention in animal models that mimic cystic fibrosis, where the effects of *P. aeruginosa* infection alone have received much attention [174,175,176,177,178,179,180,181,182,183,184,185,186,187,188,189,190,191]. In patients, this has been mostly studied in cystic fibrosis. In most cystic fibrosis studies [192,193,194], with exceptions [195], *P. aeruginosa* infection has preceded *A. fumigatus* infection, or *A. fumigatus* is commonly seen with *P. aeruginosa*, which might suggest that *P. aeruginosa* predisposes to *A. fumigatus* establishing itself as an infection, or that the pathologic processes in cystic fibrosis, having advanced as a result of repeated insults, provides a welcome milieu for *A. fumigatus* infection. Studies have indicated eradicating *P. aeruginosa* from cystic fibrosis lungs is associated with increased risk of acquiring *A. fumigatus* [196,197], although others have suggested the opposite [198]. We have indicated the *A. fumigatus* isolates in cystic fibrosis do not differ in their susceptibility to *P. aeruginosa* inhibitory factors compared to other isolates. [21]. However, despite *P. aeruginosa* more commonly preceding *A. fumigatus*, studies have shown that once *A. fumigatus* infection has become established, more episodes of *P. aeruginosa* infection will follow than would otherwise be the case [195]. Again, whether this predisposition is cause or effect is not clear. There is also good evidence that the co-infection accelerates cystic fibrosis lung disease progression [31].

## 12. Conclusions

*P. aeruginosa* and *A. fumigatus* interactions play a crucial role in the pathology of cystic fibrosis and less defined roles in immunocompromised patients. Such interactions alter the shared environmental niche and can be beneficial or inhibitory for both the species and can result in undesired effects on the mammalian host. Intermicrobial competitions and metabolite exchange between *P. aeruginosa* and *A. fumigatus* have long been studied. Stronger emphasis has been placed on co-culture experiments of *Pseudomonas* and *Aspergillus* species, which resulted in discoveries with potential therapeutic significance. It is our belief that *Pseudomonas*–*Aspergillus* interaction continues to be a promising field for mycology research and that more mycologists will be eventually attracted to these studies. This review revisits the quorum-sensing-regulated virulence factors of *P. aeruginosa* and describes how these factors affect and interact with the opportunistic fungi *A. fumigatus*. We hope that this review will be helpful to re-emphasize the complex and sophisticated nature of the *Pseudomonas–Aspergillus* interaction and indicate new avenues for future mycology studies. Better understanding of this interaction could suggest avenues for therapeutic intervention, for the benefit of the host.
